# Supplementary material for: The exocyst is an insulin-sensitive regulator of amyloid precursor protein trafficking and amyloid-beta generation in neurons
Source: bioRxiv. 2026 Apr 17:2026.04.14.717551. Preprint. [Version 1] doi: 10.64898/2026.04.14.717551 (PMC13104883; doi:10.64898/2026.04.14.717551)

bioRxiv preprint doi: https://doi.org/10.64898/2026.04.14.717551; this version posted April 17, 2026. The copyright holder for this preprint (which was not certified by peer review) is the author/funder, who has granted bioRxiv a license to display the preprint in perpetuity. It is made available under aCC-BY 4.0 International license.

# SUPPLEMENTAL INFORMATION

## SUPPLEMENTAL EXTENDED METHOD

### Microscopy and Image Acquisition

The following equipment were used to obtain high resolution images from Immunofluorescence samples. Immunofluorescence samples were imaged using a Leica SP8 inverted confocal microscope equipped with appropriate laser lines and a 63× oil-immersion objective. Images were acquired using Leica LAS X software with sequential channel acquisition to minimize spectral overlap. Acquisition parameters such as laser power, detector gain, and pinhole size, were kept constant across experimental conditions within each experiment. Z-plane images were utilized for represented images of samples unless otherwise specified, then Z-plane images were selected at maximum intensity projections. Total internal reflection fluorescence (TIRF) microscopy was performed using a Leica Thunder Live Cell 3D microscope under controlled environmental conditions (37 °C, 5% $CO_2$), with identical acquisition settings applied across conditions. Image processing was limited to linear adjustments of brightness and contrast applied uniformly across entire images.

### Data and Materials Availability

All materials including raw image files and specific protocols are available from the corresponding author upon request.

bioRxiv preprint doi: https://doi.org/10.64898/2026.04.14.717551; this version posted April 17, 2026. The copyright holder for this preprint (which was not certified by peer review) is the author/funder, who has granted bioRxiv a license to display the preprint in perpetuity. It is made available under aCC-BY 4.0 International license.

**Supplemental Figures:**

**Figure S1. Proteomics screen quality control and representative groups of cell surface proteins unchanged in differential abundance after ES2 treatment.**

A. Violin plot showing consistent global normalization and distribution of log2- normalized protein intensities across each samples per treatment (red: untreated samples, blue: ES2-treated samples).

B. Principal component analysis of log2-normalized protein intensities shows clear distinction between treated (blue) and untreated samples (red), with PC1 and PC2 explaining 61% of the total variance, indicating that the treatment is a major contributor to proteomic variation.

C. Normalized intensity of biotinylated representative cell adhesion proteins, such as CDH2, NRCAM, CADM4, with most showing no significant changes after ES2 treatment.

bioRxiv preprint doi: https://doi.org/10.64898/2026.04.14.717551; this version posted April 17, 2026. The copyright holder for this preprint (which was not certified by peer review) is the author/funder, who has granted bioRxiv a license to display the preprint in perpetuity. It is made available under aCC-BY 4.0 International license.

D. Normalized intensity of biotinylated representative plasma membrane signaling receptors, such as RET, NTRKs, IR, and other related neurotrophic proteins, each showing no significant changes after ES2 treatment.

E. Normalized intensity of biotinylated representative neuronal synaptic proteins, such as SNAP-25, SYNGR1/2, NRXN1, each showing no significant changes after ES2 treatment.

Comparison between two samples/conditions was performed using an unpaired Student's t-test. Group values were compared with one-way ANOVA and corrected via post-hoc Tukey test. Significance value with ±SD shown as *($p<0.05$), **($p<0.01$), ***($p<0.001$), **** ($p<0.0001$), and ns (not significant).

**Figure S2. Validation of exocyst gene knockdown and fluorescently-labeled APP and EXOC5 transgenes in SH-SY5Y cells.**

A. Representative immunoblot image showing EXOC5 protein knockdown in two cell lines (shExoc5#1 and shExoc5#2).

B. Quantitative real time PCR measurements demonstrated reduced exocyst gene expression in SH-SY5Y(mutAPP) cells after transfection with siRNAs against the individual eight exocyst genes.

C. Schematic representation of three fluorescent transgene constructs co-expressing mutAPP fused with mScarlet on the C-terminus, and either EXOC1, EXOC4, or EXOC7 fused with mNeonGreen on the C-terminus.

D. Validation of mutAPP-mScarlet expression in the three generated transgenic SH-SY5Y cell lines through immunoblotting with anti-RFP antibody.

E. Validated expression of exocyst subunits (EXOC1,4,7) fused to mNeonGreen in the three generated transgenic SH-SY5Y cell lines through immunoblotting with anti-mNeonGreen and anti-exocyst antibodies.

bioRxiv preprint doi: https://doi.org/10.64898/2026.04.14.717551; this version posted April 17, 2026. The copyright holder for this preprint (which was not certified by peer review) is the author/funder, who has granted bioRxiv a license to display the preprint in perpetuity. It is made available under aCC-BY 4.0 International license.

Significance value with ±SD shown as *(p<0.05), **(p<0.01), ***(p<0.001), **** (p<0.0001), and ns (not significant).

**Figure S3. Chemically inhibiting exocyst did not affect APP trafficking into the presynapses of mouse primary hippocampal neurons, marked by Bassoon (BSN) immunostaining.**

A. Representative confocal images illustrating APP (red) and Bassoon (BSN) (green) overlap (yellow) and distribution within the AIS region identified by ANKG (gray) with and without ES2. Nuclei were counterstained using DAPI (blue). Scale bar (black) = 10 µm

B. PCC analysis measuring the linear relationship between APP and ANKG immunostained regions showed significant reduction of APP puncta within the AIS region after ES2 treatment as compared to the untreated group.

C. PCC analysis of APP and BSN immunostained regions showed no significant difference between untreated and ES2 treated groups.

Statisfical analyses between two groups was done using a Student’s T-Test. Significance value with ±SD shown as *(p<0.05), **(p<0.01), ***(p<0.001), **** (p<0.0001), and ns (not significant).Dots in graphs represent multiple neurons in 1 microscopic field of biological replicates of n=3.

**Figure S4. Initial characterization and functional experiments of stable pHluorin-GLUT4-mScarlet expressing SH-SY5Y cells during insulin starvation and insulin treatments**

A. Quantification of EXOC5-APP, EXOC5-EXOC7, and EXOC5-GLUT4 PLA signal in differentiated SH-SY5Y neurons after 2 hours of insulin starvation and with (+Insulin) or without (-Insulin) 15 minutes of 100 nM insulin addback. The results shown here parallels the results demonstrated in mouse pHCN (Figure 6)

bioRxiv preprint doi: https://doi.org/10.64898/2026.04.14.717551; this version posted April 17, 2026. The copyright holder for this preprint (which was not certified by peer review) is the author/funder, who has granted bioRxiv a license to display the preprint in perpetuity. It is made available under aCC-BY 4.0 International license.

B-C. Fluorescent microplate reading of differentiated SH-SY5Y neurons stably expressing the pHluorin-GLUT4-mScarlet fusion protein, confirming increasing both pHluorin and mScarlet signal with increasing cell seeding density.

D. Fluorescent microplate reading of differentiated SH-SY5Y neurons stably expressing pHluorin-GLUT4-mScarlet showed pHluorin signal decreased when the pH of the cell medium was lowered, confirming pH sensitivity of pHluorin.

E. Fluorescent microplate reading of differentiated SH-SY5Y neurons stably expressing pHluorin-GLUT4-mScarlet showed pHluorin/mScarlet RFU decreased with longer insulin starvation times.

F. Comparison of before and after 15 minutes of 100nM insulin addback after varying lengths of insulin starvation.  Longer starvation periods yielded greater significant increases after insulation stimulation. Statistical analysis was completed using a paired T-Test as the same biological replicates during starved conditions were measured after insulin treatment.

Significance value with ±SD shown (A), ±SEM (B-F), as *(p<0.05), **(p<0.01), ***(p<0.001), **** (p<0.0001), and ns (not significant).

**Supplementary Table 1: Primary and Secondary Antibodies used in this study**

| Target | Antibody/Clone (mono, poly) | Host | Application | Dilution | Vendor | Catalog # |
|---|---|---|---|---|---|---|
| β-actin | Monoclonal | Mouse | WB | 1:000 | ProteinTech | 5606 |
| ANKG | Polyclonal | Chicken | IF | 1:500 | Novus | NBP3-05549-50ul |
| APP | Monoclonal | Rabbit | IF, WB | 1:100, 1:1000 | Abcam | ab32136 |
| ATG5 | Monoclonal | Rabbit | WB | 1:1000 | Cell Signaling | D5F5U |
| BSN | Monoclonal | Mouse | IF | 1:100 | Abcam | ab82958 |
| EXOC1 | Polyclonal | Rabbit | WB | 17:40 | ProteinTech | 11690-1-AP |
| EXOC4 | Monoclonal | Mouse | WB | 1:1000 | Enzo | ADI-VAM-SV016 |
| EXOC5 | Monoclonal | Mouse | IF, WB | 1:100, 1:1000 | Santa Cruz | Sc-514802 |

bioRxiv preprint doi: https://doi.org/10.64898/2026.04.14.717551; this version posted April 17, 2026. The copyright holder for this preprint (which was not certified by peer review) is the author/funder, who has granted bioRxiv a license to display the preprint in perpetuity. It is made available under aCC-BY 4.0 International license.

| | | | | | | |
|---|---|---|---|---|---|---|
| EXOC7 | Polyclonal | Rabbit | WB | 1:1000 | ProteinTech | 12014-1-AP |
| GLUT4 | Monoclonal | Mouse | IF, WB | 1:100, 1:500 | Santa Cruz | Sc-53566 |
| LAMP1 | Monoclonal | Rabbit | WB | 1:1000 | Cell Signaling | 9091 |
| LC3A/B | Monoclonal | Rabbit | WB | 1:1000 | Cell Signaling | 4445 |
| MAP2 | Polyclonal | Chicken | IF | 1:500 | Abcam | ab5392 |
| RAB11 | Monoclonal | Rabbit | WB | 1:1000 | Cell Signaling | 5589S |
| RAB5 | Monoclonal | Mouse | IF | 1:1000 | Cell Signaling | 46449S |
| RFP | Monoclonal | Mouse | WB | 1:1000 | Chromotek | 6g6 |
| Synaptophysin | Polyclonal | Goat | IF | 1:100 | R&D Systems | AF5555 |
| TAU | Polyclonal | Chicken | IF, WB | 1:500 | Abcam | ab75714 |
| **Selected Secondary Antibodies** | | | | | | |
| Antibody | Host: Target | Application | | Dilution | Vendor | Catalog # |
| Alexa Fluor 488 (green)- Secondary Antibody | Goat: Chicken | IF | | 1:1000 | ThermoFisher Scientific | A32931 |
| Alexa Fluor 594 (red)- Secondary Antibody | Goat: Chicken | IF | | 1:1000 | ThermoFisher Scientific | A-11042 |
| Donkey Anti-Goat IgG H&L (Alexa Fluor® 647) preabsorbed | Donkey: Goat | IF | | 1:1000 | Abcam | ab150135 |
| Licor Secondary (red) | Goat: Rabbit | WB | | 1:10,000 | Licor | 926-68071 |
| IRDye® 680RD IgG Secondary Antibody | Donkey: Mouse | WB | | 1:10,000 | Licor | 926-68072 |
| IRDye® 800CW IgG Secondary Antibody | Goat: Rabbit | WB | | 1:10,000 | Licor | 926-32211 |

## Supplemental Figure 1

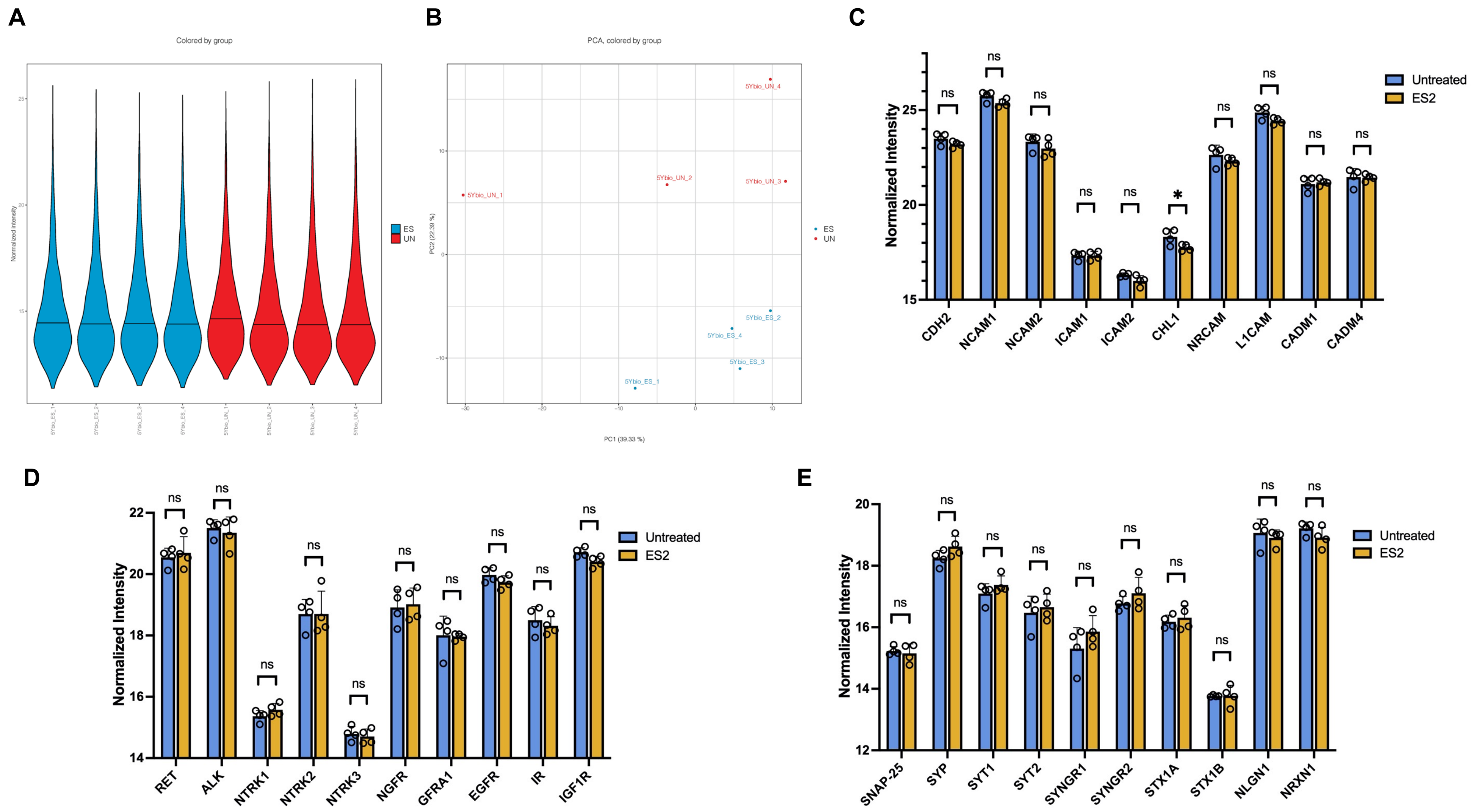

## Supplemental Figure 2

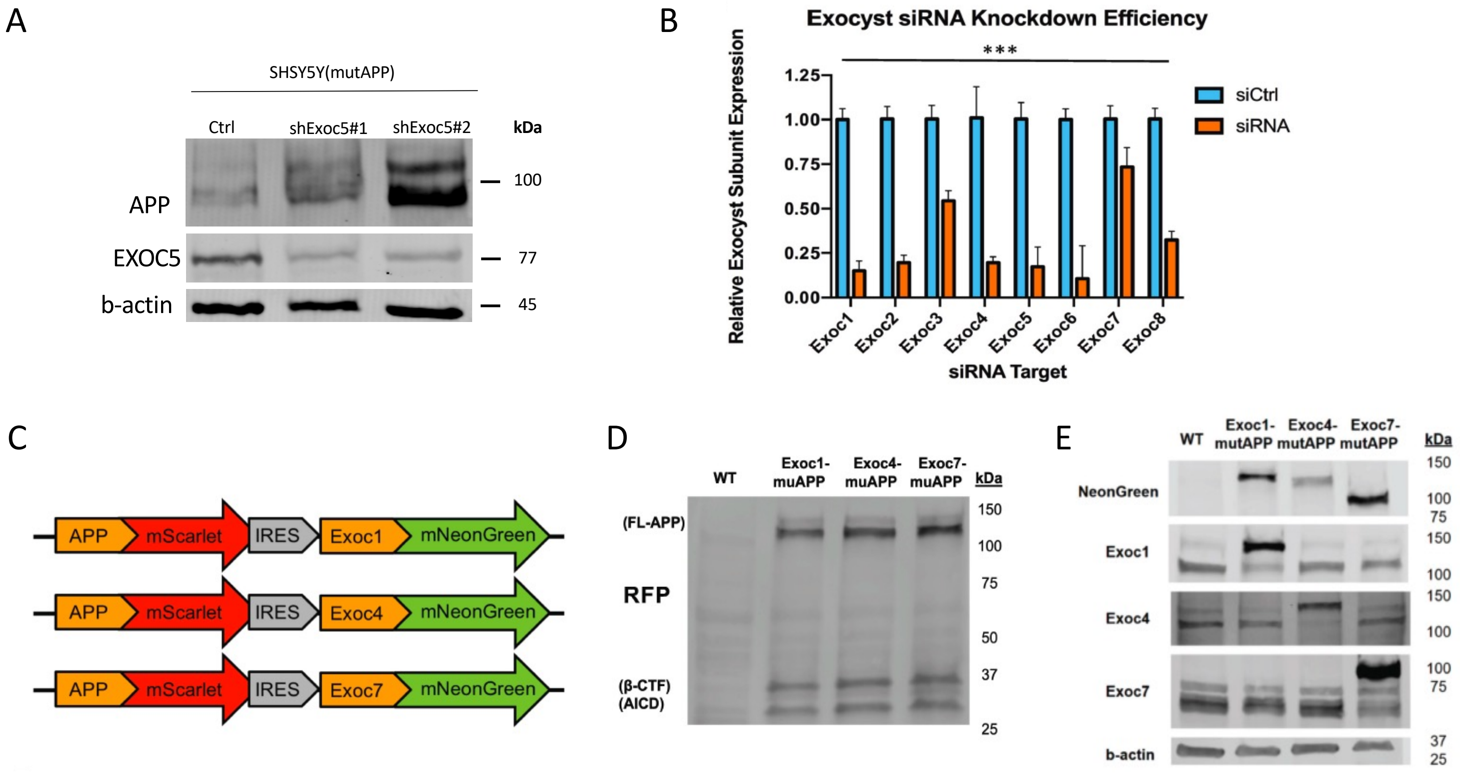

# SUPPLEMENTAL Figure 3

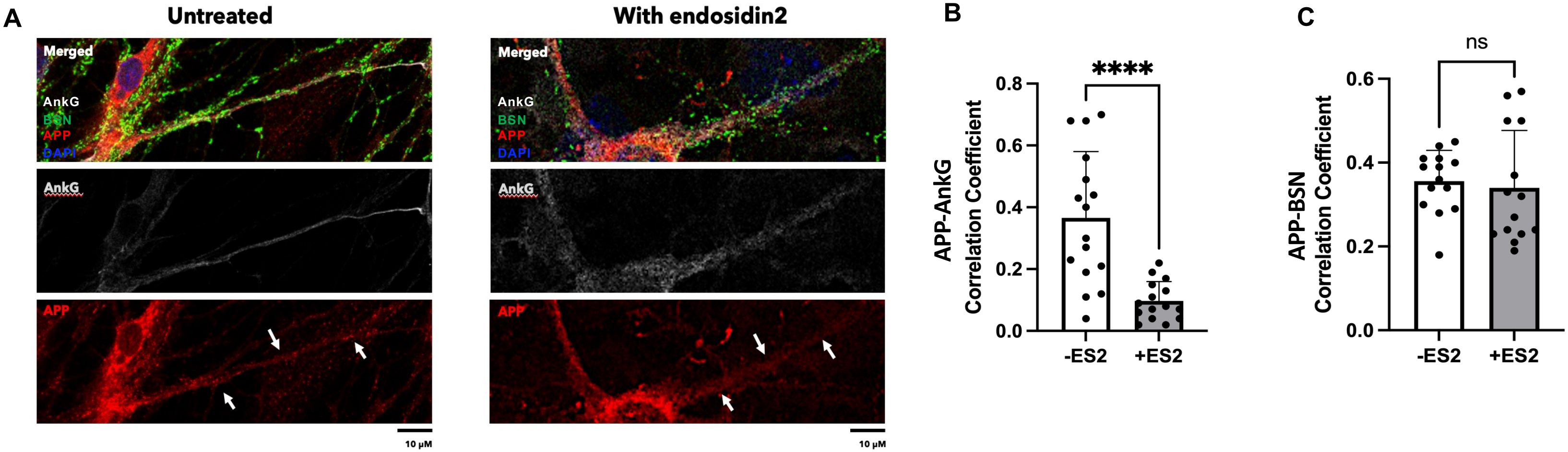

Supplemental Figure 4

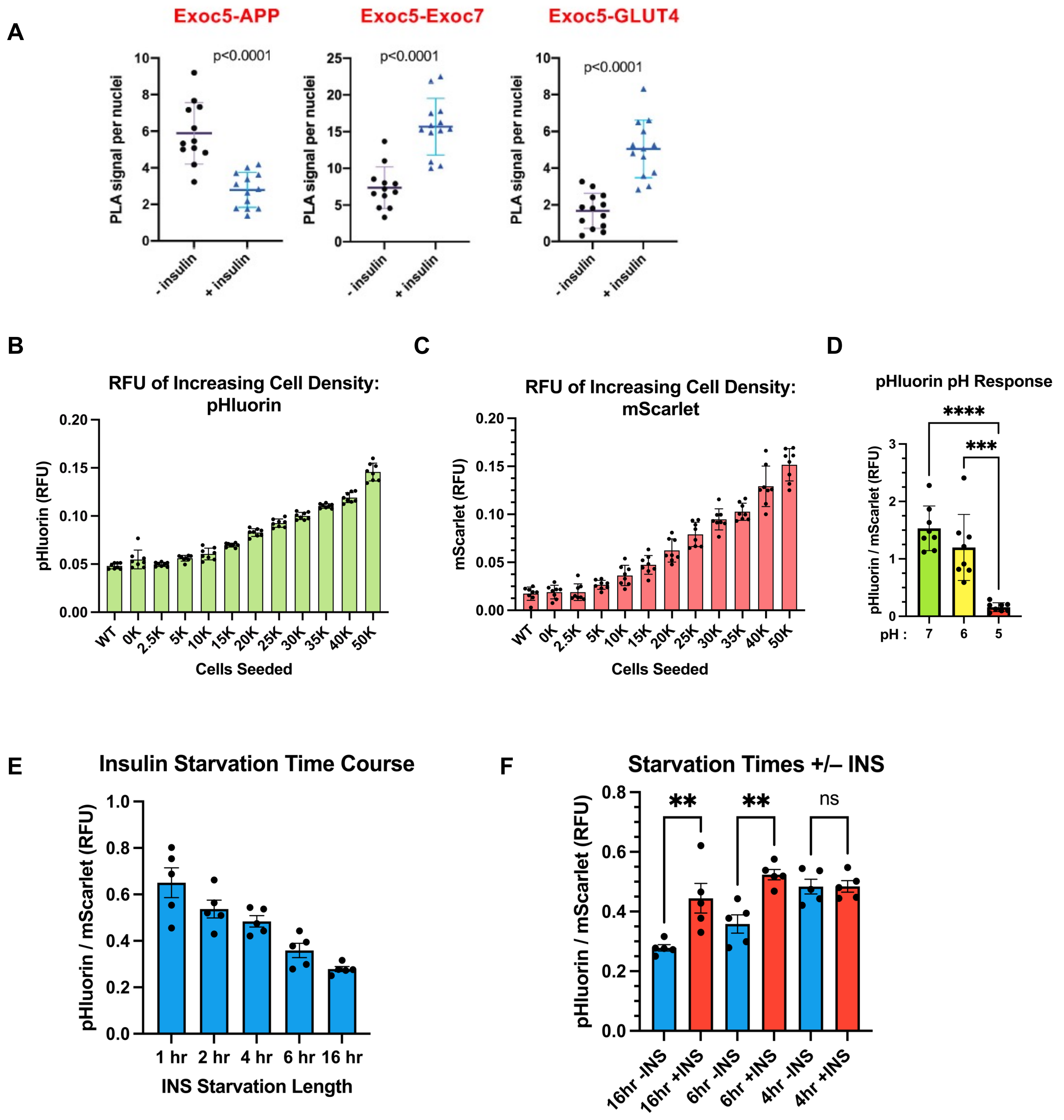

Supplement: Supplement 1 [file NIHPP2026.04.14.717551v1-supplement-1.pdf]
